# The Yin-Yang of myeloid cells in the leukemic microenvironment: Immunological role and clinical implications

**DOI:** 10.3389/fimmu.2022.1071188

**Published:** 2022-12-01

**Authors:** Fábio Magalhães-Gama, Fabíola Silva Alves-Hanna, Nilberto Dias Araújo, Mateus Souza Barros, Flavio Souza Silva, Claudio Lucas Santos Catão, Júlia Santos Moraes, Izabela Cabral Freitas, Andréa Monteiro Tarragô, Adriana Malheiro, Andréa Teixeira-Carvalho, Allyson Guimarães Costa

**Affiliations:** ^1^ Diretoria de Ensino e Pesquisa, Fundação Hospitalar de Hematologia e Hemoterapia do Amazonas (HEMOAM), Manaus, Brazil; ^2^ Programa de Pós-Graduação em Ciências da Saúde, Instituto René Rachou - Fundação Oswaldo Cruz (FIOCRUZ) Minas, Belo Horizonte, Brazil; ^3^ Grupo Integrado de Pesquisas em Biomarcadores de Diagnóstico e Monitoração, Instituto René Rachou – FIOCRUZ Minas, Belo Horizonte, Brazil; ^4^ Programa de Pós-Graduação em Imunologia Básica e Aplicada, Instituto de Ciências Biológicas, Universidade Federal do Amazonas (UFAM), Manaus, Brazil; ^5^ Programa de Pós-Graduação em Ciências Aplicadas à Hematologia, Universidade do Estado do Amazonas (UEA), Manaus, Brazil; ^6^ Escola de Enfermagem de Manaus, UFAM, Manaus, Brazil

**Keywords:** leukemia, neutrophils, macrophages, myeloid-derived suppressor cells, immune response, tumor microenvironment, immunotherapy

## Abstract

The leukemic microenvironment has a high diversity of immune cells that are phenotypically and functionally distinct. However, our understanding of the biology, immunology, and clinical implications underlying these cells remains poorly investigated. Among the resident immune cells that can infiltrate the leukemic microenvironment are myeloid cells, which correspond to a heterogeneous cell group of the innate immune system. They encompass populations of neutrophils, macrophages, and myeloid-derived suppressor cells (MDSCs). These cells can be abundant in different tissues and, in the leukemic microenvironment, are associated with the clinical outcome of the patient, acting dichotomously to contribute to leukemic progression or stimulate antitumor immune responses. In this review, we detail the current evidence and the many mechanisms that indicate that the activation of different myeloid cell populations may contribute to immunosuppression, survival, or metastatic dissemination, as well as in immunosurveillance and stimulation of specific cytotoxic responses. Furthermore, we broadly discuss the interactions of tumor-associated neutrophils and macrophages (TANs and TAMs, respectively) and MDSCs in the leukemic microenvironment. Finally, we provide new perspectives on the potential of myeloid cell subpopulations as predictive biomarkers of therapeutical response, as well as potential targets in the chemoimmunotherapy of leukemias due to their dual Yin-Yang roles in leukemia.

## Introduction

Leukemias correspond to a heterogeneous group of hematological malignancies that are characterized by a blockage in maturation and/or proliferation of hematopoietic cells of the myeloid or lymphoid lineage, which can be divided into acute or chronic forms (1). As a hallmark, acute leukemias have a deep blockage in hematopoietic differentiation, which results in overproduction of leukemic blasts, while chronic leukemias are characterized by the excess production of partially mature differentiated cells (2, 3). Collectively, leukemia affects individuals of all ages; however, in adults, the most common types of leukemia are acute myeloid (AML), chronic myeloid (CML), and chronic lymphocytic leukemia (CLL); while acute lymphoblastic leukemia (ALL) affects mainly children and represents the most frequent pediatric cancer in the world (4–7).

As in other types of cancer, one of the most important mechanisms by which leukemic cells (LCs) promote their development is the generation of an immune microenvironment that inhibits and impairs antitumor responses (8–10). The leukemic microenvironment represents a highly complex cellular compartment that comprises diverse cell populations, which include non-hematopoietic stromal cells, vascular endothelial cells, and innate and adaptive immune cells (11–13). During the process of leukemogenesis, stromal cells within the medullary compartment keep premalignant cells under control; however, as LCs develop, they initiate intense crosstalk with the full range of adjacent cells (11, 14). This dynamic crosstalk between LCs and components of the medullary compartment, in addition to contributing to the increase in the availability of growth and survival factors, also promotes the recruitment and polarization of immune cells into the leukemic niche (10, 11).

Among the immune cells that can infiltrate the leukemic microenvironment are myeloid cell populations, which correspond to a heterogeneous cell group of the innate immune system, including tumor-associated neutrophils (TANs), tumor-associated macrophages (TAMs), and myeloid-derived suppressor cells (MDSCs) (15). These cell populations have received great attention over the last decade because they are recruited to tumor niches on a large scale, and can act as critical components for the suppression of innate and adaptive immune responses, and also in the reduction of efficacy of immunotherapeutic approaches (16, 17). At the same time, due to their high plasticity, the subpopulations of tumor-associated neutrophils and macrophages have been shown to represent important antitumor effector cells, which can eliminate malignant cells and activating other cytotoxic effector cells. This highlights their ability to be reprogrammed into an antitumor phenotype in order to maximize tumoricidal defenses (18–22).

In this context, the advent of a “Yin-Yang” role for these innate immune cell populations in the field of leukemias remains poorly understood, despite their multiple functions and effects in solid tumors being well understood. Thus, in this review, we describe the main aspects related to myeloid cell populations, including their biological characteristics, immunological mechanisms, involvement in tumor-targeted responses, clinical implications, as well as their impact on the leukemic microenvironment. Finally, we provide a new perspective on the importance of investigating the wide diversity of these cell types in hematological malignancies and highlight their potential as prognostic biomarkers and relevant therapeutical targets, which can complement the treatment of patients with leukemia.

## Tumor-associated neutrophils (TANs)

Neutrophils are the most abundant leukocytes in the blood and are considered the first line of defense during inflammatory and infectious processes, in which they release a range of inflammatory mediators (23–25). Besides their classical roles in antimicrobial responses, neutrophils can be found in tumor infiltrates, and are named tumor-associated neutrophils (TANs). They also exhibit great plasticity that allows them to perform diverse and often opposite functions (26–29). TANs can display favorable or harmful responses to the host depending on their polarization, which, in a simplified manner, can be classified into two distinct phenotypes, N1 (tumor suppressor) and N2 (tumor promoter) (30, 31).

**Figure 1:**
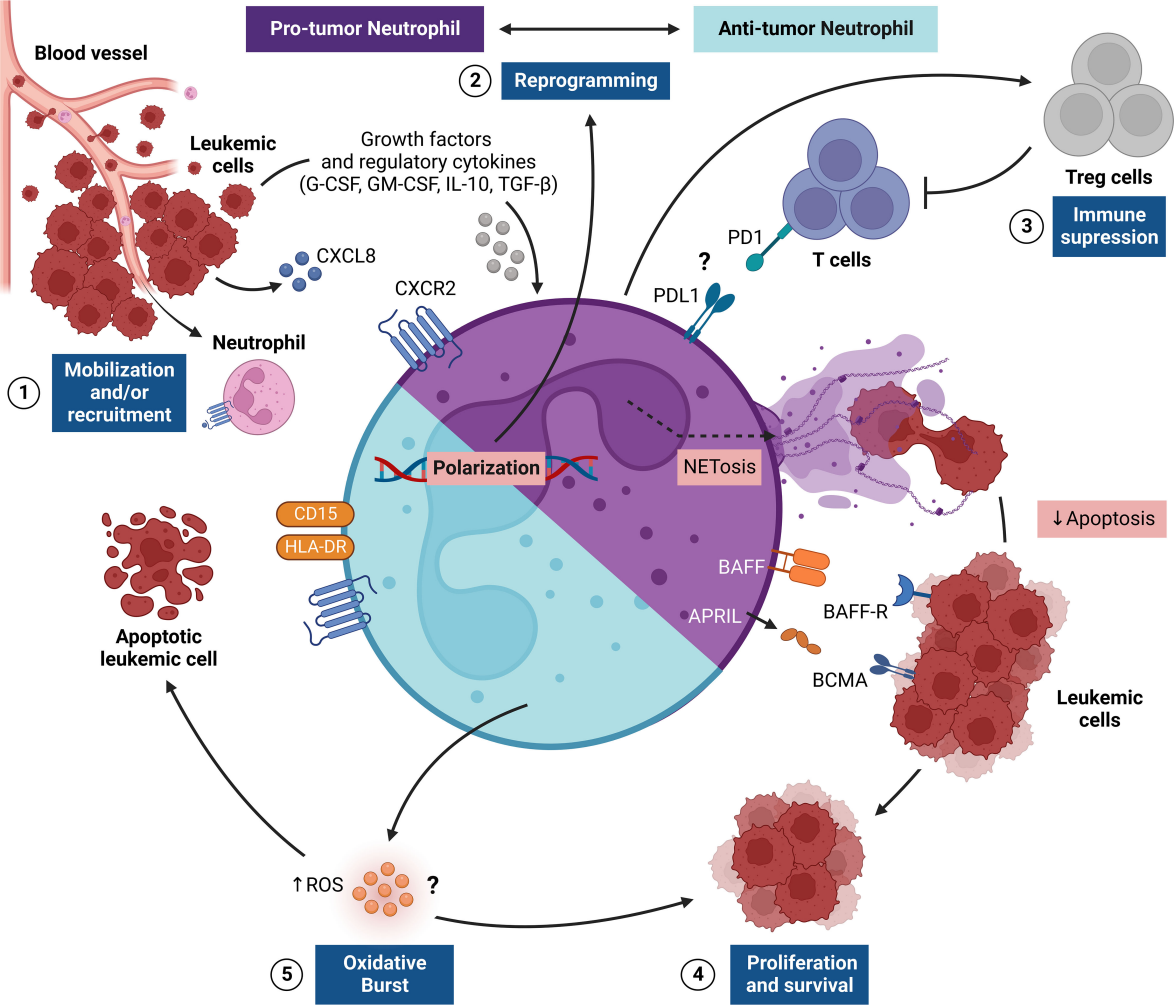
Regulation of TAN responses in the leukemic microenvironment. The figure shows the pro-tumor and antitumor activities of tumor-associated neutrophils (TANs), which provide critical signals for leukemic progression. This network of interactions between TANs, leukemic cells (LCs) and other immune cells [e.g., regulatory T cells (Treg)] results in four main events: [1] recruitment and/or mobilization of neutrophils *via* CXCL8/CXCR2 to the leukemic microenvironment, where LCs promote the survival of TANs through the release of G-CSF and GM-CSF; [2] reprogramming of TANs to become pro-tumor (N2) or antitumor (N1) cells through stimulation of the leukemic microenvironment through the cytokines IL-10 and TGF-β; [3] immunosuppression promoted by N2 TANs that can preferentially recruit Treg cells and can suppress T cell effector responses, apparently *via* a PD1-PDL1-mediated pathway; [4] LC proliferation and survival in the leukemic microenvironment regulated by a range of surface molecules (e.g., BAFF and APRIL) expressed by N2 TANs, which also release NETs, which, in turn, delay LC apoptosis; [5] In another scenario, the oxidative burst triggered by N1 TANs can promote the release of reactive oxygen species (ROS) that control proliferation or mediate the killing of LCs. APRIL, A proliferation-inducing ligand; BAFF, B-cell activating factor; G-CSF, granulocyte colony-stimulating-factor; GM-CSF, Human granulocyte-macrophage colony-stimulating factor; NETs, ​​Neutrophil extracellular traps; PD1, Programmed cell death protein 1; PDL1, Programmed cell death-ligand 1; TGF-β, Transforming growth factor-β.

N1 TANs are usually found in the early stages of the tumorigenic process, and act in the recruitment and activation of cytotoxic T cells and T helper (Th) cells (32–35). They emerge mainly from IFN-β–mediated stimuli, and are characterized by the high expression of immuno-activating cytokines and chemokines, such as TNF, IL-12, CCL-3, CXCL-9, and CXCL-10, in addition to the expression of ICAM-1, FAS and low levels of Arginase-1 (26, 36, 37). As for N2 TANs, they appear in later stages, inhibit effector responses and preferentially recruit regulatory T cells (32–35). They differ mainly after stimulation mediated by TGF-β and IL-35 and, unlike N1 TANs, they are characterized by the overexpression of Arginase-1, the presence of protease-enriched granules, such as neutrophil elastase (NE), cathepsin G (CG) and matrix metalloproteinases (MMPs), in addition to the production of a wide range of chemokines that include CCL-2, CCL-5, and CXCL-8 (**Figure 1**) (26, 34, 36, 38). Notably, TANs are usually distinguished based on their anti- or pro-tumor functions, as mentioned earlier (33). In terms of expression of intracellular and extracellular markers, the literature generally mentions CD11b, HLA-DR^lo/int^, CD15^hi^, CD16^hi,^ and CD66, with varying expression of CD33 and Arginase-1 (16, 36, 39–41). In addition, recent studies have characterized anti- or pro-tumor TAN populations based on the presence and expression of CD54^+^, HLA-DR^+^, CD86^+^ and CD15^hi^; and CD170^hi^ and PD-L1^+^ (42).

Traditionally, neutrophils act as key elements in the immune system, and play a fundamental role in the immune response through a range of mechanisms, which include: phagocytosis and generation of reactive oxygen species (ROS); antibody-dependent cell-mediated cytotoxicity (ADCC); degranulation and release of proteases; immune cell modulation; and release of extracellular neutrophil traps (NETs), which consists of modified chromatin decorated with bactericidal proteins from granules and cytoplasm (37, 43–46). However, in the tumor context, the effector function of these cells is usually modified to contribute to cancer cell progression through processes such as extracellular matrix remodeling, angiogenesis and lymphangiogenesis, immunosuppression, and promotion of cancer cell invasion and metastasis (47–52). Given these characteristics, besides the fact that they represent up to 70% of circulating leukocytes and are present in large numbers in the tumor microenvironment (TME), TANs are being increasingly investigated in many types of cancer (53–57).

In the context of hematological malignancies, the role of neutrophils in the TME is poorly studied. In the field of leukemias, the role of TANs has been investigated mainly in CLL, in which it was observed that *in vitro* stimulated neutrophils were more prone to release of NETs, which in turn, have been shown to delay apoptosis and increase the expression of activation markers on LCs (58). This increased susceptibility is driven by the CXCL-8 chemokine, which exhibited significantly elevated levels in the plasma of CLL patients and correlated with the ability to release NETs after neutrophil activation. Of note, plasma from CLL patients has been shown to increase CXCR2 expression in neutrophils, thus allowing CXCL-8-mediated activation; however, the plasma factors responsible for these upregulatory effects remain unknown (58).

Initial observations suggested the possibility that LCs could be driving the maintenance and regulation of TANs to promote leukemic progression. Nonetheless, *via* further investigations, it was observed that LCs promoted TANs survival through release of G-CSF/GM-CSF and induced their reprogramming through IL-10 and TGF-β cytokines in CD16^high^ CD62L^dim^ subsets, which are capable of significantly suppressing the effector functions of T cells, such as proliferation and IFN-γ production (59). Moreover, it has been shown that patients with CLL exhibit increased amounts of circulating immunosuppressive neutrophils (CD16^high^ CD62L^dim^), and that, depending on the disease stage in the intermediate or advanced state, there is a strong trend toward an increase in the frequency of this TANs subset (59). Such observations reinforce the ability of LCs to induce neutrophil reprogramming to the N2 phenotype and suggest their association with a poor prognosis in CLL patients.

CLL is usually accompanied by immunosuppression and susceptibility to infections (60). Studies carried out seeking to compare the characteristics of neutrophils from patients with CLL who had infections with non-infected patients observed a decrease in chemotaxis and oxidative burst in neutrophils in infected patients (60, 61). Similar results were seen in pediatric patients with ALL, in which oxidative burst was shown to be significantly suppressed in these patients at the time of diagnosis and after remission chemotherapy (62). In addition, studies have also shown that neutrophils from CLL patients have impaired bactericidal activity (60, 63). These data are indicative that neutrophils derived from patients with CLL and ALL tend to lose their effector functions while acquiring pro-tumorigenic capabilities, thus polarizing to the N2 phenotype.

Significant differences in phenotypic characteristics of neutrophils were found in another study that evaluated the expression of receptors and adhesion molecules associated with inflammation. An increased frequency of CD54^+^ and CD64^+^ (FcγRI) neutrophils was observed, along with an increase in ROS-generating activity (64). It has been suggested that the activation phenotype of circulating neutrophils could be the result of a systemic inflammatory environment, and is reinforced by high levels of TNF, which would contribute to the survival and proliferation of LCs, while the increase in oxidative potential could be related to chronic activation of the immune system. Interestingly, despite the activation status, neutrophils from CLL patients failed to mount a standard inflammatory response, thus highlighting an unusual activation phenotype with suppressed functional features that possibly comes from stimuli from the leukemic microenvironment (64).

Indeed, stromal cells from the leukemic microenvironment have been shown to interact substantially with neutrophils (65, 66). A study realized in an Eμ-TCL1 CLL murine model indicated that stromal cells from red pulp and the marginal zone of spleens presented LCs infiltrate, overexpressed genes related to neutrophil chemotaxis, including the chemotactic factors S100A8 and S100A9 (MRP8 and MRP14, respectively), the chemokine receptor CXCR2, the pro-inflammatory cytokine IL-1β, and integrin CD11b (Itgam) (65, 66). In addition, neutrophils infiltrated in the leukemic niche were observed to secrete survival cytokines, including APRIL and BAFF, which are important for B cell proliferation. Finally, the neutrophil depletion in Eμ-TCL1 mice, with already developed CLL, demonstrated a reduction in the leukemic burden in spleens, clearly showing that neutrophils support leukemia progression in Eμ-TCL1 mice (65, 66).

Despite all the data presented, little is known about the influence of neutrophils on the development of the antileukemic immune response, especially if we consider the other types of leukemia, which practically remain unexplored. This reinforces the need for investigations into the mechanisms by which neutrophils contribute to disease pathogenesis, besides the impact of their dysfunction on effective immune defense against pathogens and their potential association with the frequent occurrence of severe infections during the period during chemotherapy treatment.

## Tumor-associated macrophages (TAMs)

Macrophages are innate effector cells with high phagocytic power and exhibit high functional plasticity that is context-dependent. In other words, they appear in response to different stimuli and assume different functional, phenotypic, and morphological identities (67, 68). Overall, macrophages play a crucial role in activating innate and adaptive responses, since they are important components in defense against infections, tissue repair, antigen presentation, and subsequent initiation of T and NK cell responses in different microenvironments (68).. When detecting tissue alterations, non-activated macrophages (Mφ) assume various activation states within a broad phenotypic and functional spectrum, thus characterizing the M1-M2 macrophage polarization system, which indicates whether these innate immune cells are more pro-inflammatory or anti-inflammatory (69).

**Figure 2:**
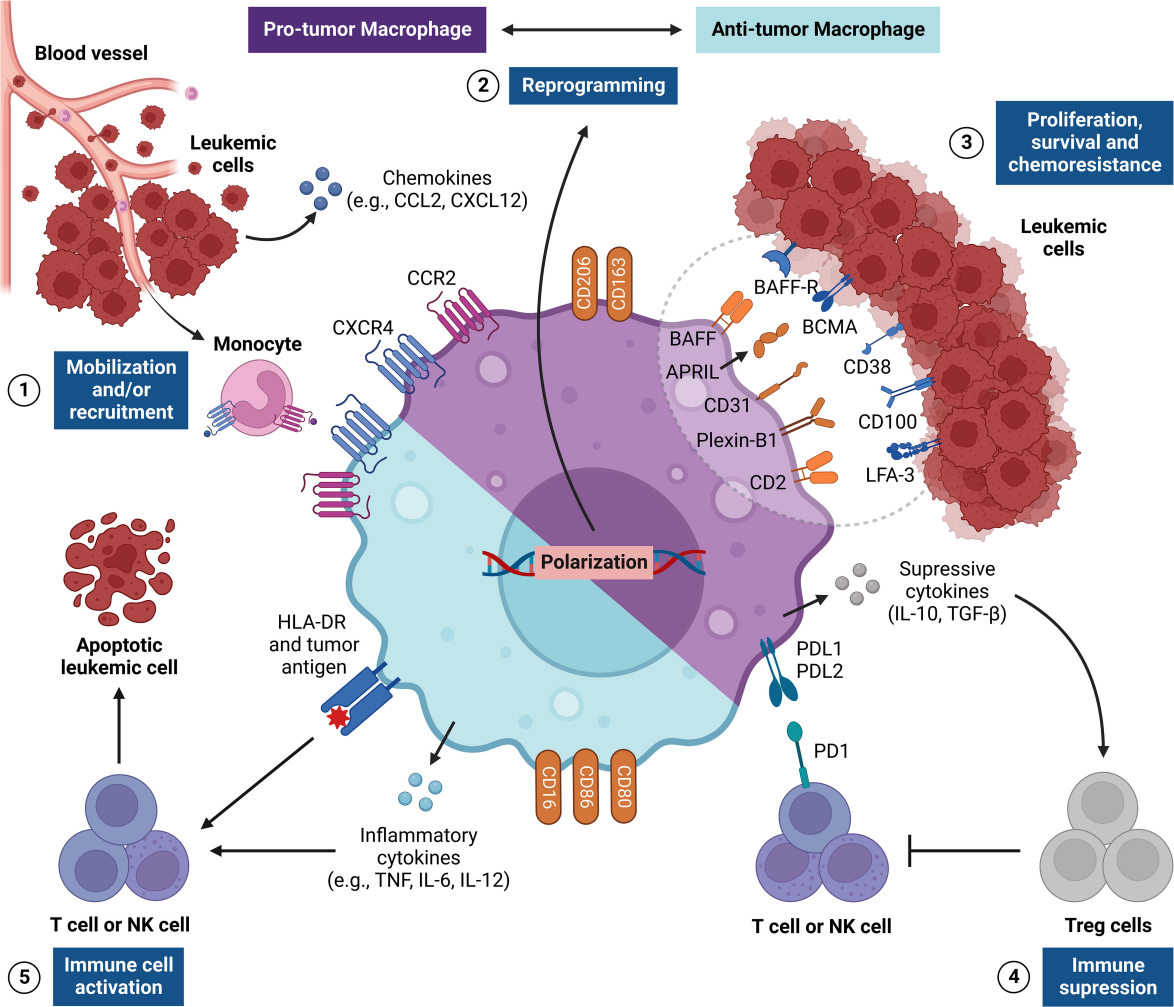
Regulation of TAM responses in the leukemic microenvironment. The figure shows the pro-tumor and antitumor activities of tumor-associated macrophages (TAMs), which are critical signs for leukemic progression. This network of interactions between TAMs, LCs and other immune cells [e.g., NK cells and Treg cells] results in five main events: [1] recruitment and/or mobilization of monocytes *via* CXCL8/CXCR2 and CCL2/CCR2 to the leukemic microenvironment, which differentiate into TAMs; [2] reprogramming TAMs to become pro-tumor (M2) or antitumor (M1) cells through many stimuli from the leukemic microenvironment, such as cytokines and cell-cell crosstalk (i.e., context dependent); [3] immune interactions with LCs within the leukemic microenvironment through a wide range of surface molecules that send many signals to stimulate the proliferation, survival and chemoresistance in LCs (e.g., BAFF, APRIL, CD31, Plexin-B1 and CD2); [4] establishment of a strongly tolerogenic environment, such as M2 TAMs, which produce many suppressive cytokines (e.g., IL-10 and TGF-β) and express immune checkpoints (PDL1 and/or PDL2), and which regulate the responses of T NK cells, and stimulate Treg cell activities; [5] establishment of an inflammatory environment, such as M1 TAMs, thus stimulating the cytotoxic activity of T and NK cells by the production of inflammatory cytokines (e.g., TNF and IL-12) and the expression of co-stimulatory molecules (e.g., CD80, CD86 and HLA-DR), which can promote antitumor responses and the death of the tumor. APRIL, A proliferation-inducing ligand; BAFF, B-cell activating factor; BAFF-R, BAFF receptor; BCMA, B-cell maturation antigen; CCR2, C-C motif chemokine receptor 2; CXCR4, C-X-C chemokine receptor 4; HLA-DR, Human leukocyte antigen DR; LFA-3, Lymphocyte function-associated antigen 3; NK, Natural killer; PD1, Programmed cell death protein 1.

M1 or ‘classically activated’ macrophages arise after stimuli mediated by IFN-γ, TNF, GM-CSF, and bacterial LPS. They are capable of producing substantial levels of pro-inflammatory cytokines, such as IL-1β, IL-6, IL-12, IL-23, and TNF (70), and express different intracellular and extracellular markers that allow their distinction, and present cells that are positive for CD68, CD11c, CD14, CD80, CD86, HLA-DR, iNOS and signal transducer and activator of transcription (STAT)-1 (71). On the other hand, M2 or ‘alternatively activated’ macrophages appear after stimuli mediated by cytokines such as IL-4 and IL-13, and are able to produce anti-inflammatory molecules such as IL-10 and TGF-β (**Figure 2**) (70). Common intracellular and extracellular markers for M2 macrophages include CD68, CD14, CD163, CD204, CD206, STAT3, STAT6, Arginase-1, VEGF and cMAF (72). It is important to note that the designation M2 covers other macrophage populations (M2a, M2b, M2c, and M2d), whose phenotypic and functional diversity remains poorly understood (72).

The different subsets of macrophages are critically involved in the progression or regression of several diseases, including cancer (73). As the tumors progress, they are accompanied by an extensively dysregulated hematopoiesis, reflecting in a continuous expansion and renewal of myeloid cells in the bone marrow (BM) and in peripheral sites, which travel between different tissues and contribute to immunosuppression or immunosurveillance (74, 75). As highly heterogeneous cells, macrophages are in continuous communication with the periphery beyond the TME. Therefore, the presence of tumor-associated macrophages (TAMs) is now seen as an important signal of the regulation of tumor immunity, since (i) TAMs infiltrate the TME to regulate tumor growth and (ii) the TME shapes the activity and functional use of TAMs to promote cancer cell metastasis and survival (76).

Many signals that shape the M1/M2 polarization vary according to the type, stage, location of the tumor, and result in different highly dynamic TAM phenotypes (77). Each macrophage is not always functionally restricted to a specific role. For example, it is important to note that IL-6 and IL-1β-producing TAMs, possibly M1-like, promote tumor growth through the production of these cytokines, which shows that M1 TAMs are not always able to precisely exert their antitumor functions during cancer progression (78–80). Further strong evidence of this is that, in some tumors, TAMs that exhibit an M2 phenotype (CD163^+^ CD206^+^) perform an activity that is equivalent to M1-like TAMs, and initiate antitumor responses (81–83). Therefore, the M1/M2 classification is too simplistic for this highly heterogeneous cell type.

Generally, TAMs have a recognized role in solid tumors, but their involvement in hematologic malignancies, such as leukemia, remains unclear. There is evidence that crosstalk between TAMs and LCs occurs at different sites during leukemia progression (60, 84–90). The fact that these cells are widely distributed in tissues suggests that TAMs travel to leukemic niches and support the proliferation of LCs, which influences the clinical outcome of the disease (90–94). These early insights are supported by several clinical and preclinical findings that have demonstrated that tumor progression affects the biodistribution of TAMs in the BM and at extramedullary sites, in which M1/M2 balance is extensively dysregulated following recruitment and reprogramming of TAMs to a pro-tumor phenotype (M2) (88, 90, 95–99).

Mobilization of TAMs to leukemic niches is supported by chemokines such as CCL2, CCL3, CCL4, and CXCL12, which regulate their biodistribution (100, 101). Among these molecules, CXCL12 promotes the mobilization of CXCR4+ TAMs to the BM microenvironment and supports M2 polarization (87, 102–104). It is important to note that CCL2 also regulates the infiltration of TAMs into the TME, which, in turn, also produces CCL2 and amplifies the mobilization of more CCR2+ macrophages to the tumor niche (104–106). Leukemia patients exhibit elevated levels of CCL2 and CXCL12 in the BM and blood, which can critically influence the activation and recruitment of TAMs (104, 107–111).

Another important point is that extramedullary sites, such as the lymph nodes, the spleen and the liver, also support the growth and metastatic spread of LCs, and macrophages act as essential stromal components in these compartments (112). Therefore, the idea that local macrophage-mediated signaling pathways are decisive elements for tumor progression is well established in the context of leukemia (91, 113, 114). In line with this, several reports have shown that LCs mobilize CCR1+ or CCR4+ TAMs in lymph nodes *via* CCL3 and CCL4 (100, 115), which in turn preserve the survival of LCs through cell-cell interactions (116). Similar events also occur in the spleen and liver of mice, in which a high accumulation of M2 TAMs is regulated by the CCR2-CCL2 axis (95, 117). On the other hand, in some cases, M1 TAMs exhibit a curious predominance in the BM and liver of these mice (118). This is evidenced when spleen-derived macrophages are cultured with LCs and promote greater tumor growth *in vitro* when compared to cultured BM-derived macrophages (99, 119), thus suggesting that different subsets of TAMs may play pleiotropic roles at different sites, since liver TAMs exhibited greater production of inflammatory factors such as CCL5, TNF, and IL-12 than BM and spleen TAMs (120, 121).

By infiltrating different leukemic niches, M2 TAMs produce soluble mediators that support LC expansion and survival (84). When monocytes from healthy donors are cultured with LCs, they differentiate into M2 and increase the resistance of LCs to apoptosis *via* CXCL12 secretion (113). Additionally, some cytokines and chemokines may also participate in this process, since M2 TAMs also secrete IL-8, IL-10, TGF-β, CCL2, CCL4, CXCL13, and other soluble mediators that are capable of inducing tumor growth *in vitro* and *in vivo* (98, 117, 121–125). In turn, LCs can produce substantial levels of IL-4, IL-10, IL-13, NAMPT, Arginase-2, and BMP-4, which regulate the pro-tumor functions of TAMs (84, 122, 123, 126).

An emerging mechanism of cell-cell communication during tumor progression is the release of exosomes, endosomal-derived vesicles, which carry different molecular components (e.g., nucleic acids, proteins, or metabolites) and are present in several biological fluids, being secreted by many cell populations, including immune and cancer cells (127, 128). These extracellular vesicles play an important role in the crosstalk between LCs and TAMs and reprogram these leukocytes into leukemia-supportive effector cells. Previous studies have reported that LC-derived exosomes are able to polarize macrophages to an immunosuppressive phenotype with enriched expression of PD-L1 on the cell surface and overproduction of IL-10 (129, 130). The high expression of PD-L1 by TAMs allows it to suppress the activity of PD1+ T cells and NK cells (11, 117).

Other receptors act to send survival signals to LCs. Studies have shown that CD163 binds to the unknown ligand on LCs, and its expression correlates with an increased leukemia burden (90, 98). In this sense, it is worth noting that CD163 induces a strong production of IL-10 by macrophages (131), in which this cytokine is a potent stimulator of malignant B cells (132, 133). In addition, M2 TAMs upregulate the expression of BAFF and APRIL, which bind to BAFF-R and BCMA expressed in LCs, respectively (134, 135). These ligands may be related to the resistance of LCs to chemotherapy and apoptosis, as previously reported (134, 136, 137). Finally, the expression of CD31 and plexin-B1 by these cells leads to an increased potential for LC survival (101, 138). This is because LCs express CD38 and CD100, which are receptors for CD31 and plexin-B1, respectively (138, 139). The CD2–LFA-3 axis was also shown to be critical in the crosstalk between M2 TAMs and LCs, as it also acts on the sending of stimulatory signals to LCs (116).

Although the functions of TAMs are often related to leukemic progression, it is still difficult to establish their precise prognostic value in leukemia. While several reports highlight their strong pro-tumor interactions through the production of soluble mediators and surface receptors, some evidence suggests that M1 TAMs can be mobilized to the BM or extramedullary sites (99, 118–121). Whether they are playing a protective role is still an open question, but strong evidence of this is that when M2 TAMs are treated with IFN-γ, they polarize to an M1 profile, inhibit their regulatory activity, and increase HLA-DR expression, CD86, and CD64, and exhibit a high antileukemic response *in vitro* (140). However, the antitumor activity of TAMs in leukemia remains poorly investigated; although, it is evident that M1 TAMs produce inflammatory factors in the TME (120, 121). The fact is that the presence of M2 TAMs is generally associated with an increased leukemic burden and, subsequently, an unfavorable prognosis (90, 118, 141). Therefore, future investigations should focus on the different macrophage subsets and their functional and prognostic relevance in different leukemia subtypes.

## Myeloid-derived suppressor cells (MDSCs)

Myeloid-derived suppressor cells (MDSCs) correspond to a phenotypically heterogeneous cell population that is derived from immature myeloid precursors of the granulocytic or monocytic lineage (16). The ontogeny process of MDSCs involves blocking differentiation in normal hematopoiesis, thus preventing their terminal differentiation, and promoting their expansion. This feature highlights their distinction from terminally differentiated, mature myeloid cells; although their distinction from neutrophils is usually a controversial topic (16, 142). However, studies have shown that these cells can also differentiate from a monocyte-like precursor of granulocytes (143). In humans, there is still a recently discovered subpopulation known as “early MDSCs”, which represent less than 5% of the MDSC population; however, little is known about their role (144). In general, as evidenced by their name, pathologically activated MDSCs exhibit strong immunosuppressive capabilities, and act as crucial drivers of an immunosuppressive microenvironment (16).

MDSCs are subdivided into two groups: polymorphonuclear MDSCs (PMN-MDSCs), which are morphologically similar to neutrophils; and monocytic MDSCs (M-MDSCs), which are morphologically similar to monocytes (142, 145). In humans, they are identified from specific cell markers; however, these are far from uniform. PMN-MDSCs are defined as CD11b^+^ CD14^-^CD15^+^/CD66^+^ cells (146–148). In turn, M-MDSCs are defined as CD14^+^ CD15^-^/CD66^-^ HLA-DR^lo/-^ (144). CD14 is a characteristic surface marker of monocytes, while HLA-DR^lo/-^ helps distinguish M-MDSCs from mature monocytes and CD15^-^ distinguishes M-MDSCs from PMN-MDSCs (149). As PMN-MDSCs are morphologically and phenotypically like classical neutrophils, the main way to differentiate them is functionally, i.e., based on their ability to suppress other immune cells, since normal neutrophils are not immunosuppressive cells (150–152). On the other hand, there is still an intense debate on the distinction between N2 TANs and PMN-MDSCs, due to shared origin, phenotypic and functional characteristics. However, despite the high similarity between these cell populations, it has recently been shown that lectin-like oxidized LDL receptor-1 (LOX-1), which is highly expressed in human PMN-MDSCs, may represent a specific marker to distinguish these cells from mature neutrophils in peripheral blood and tumor tissues (153).

**Figure 3:**
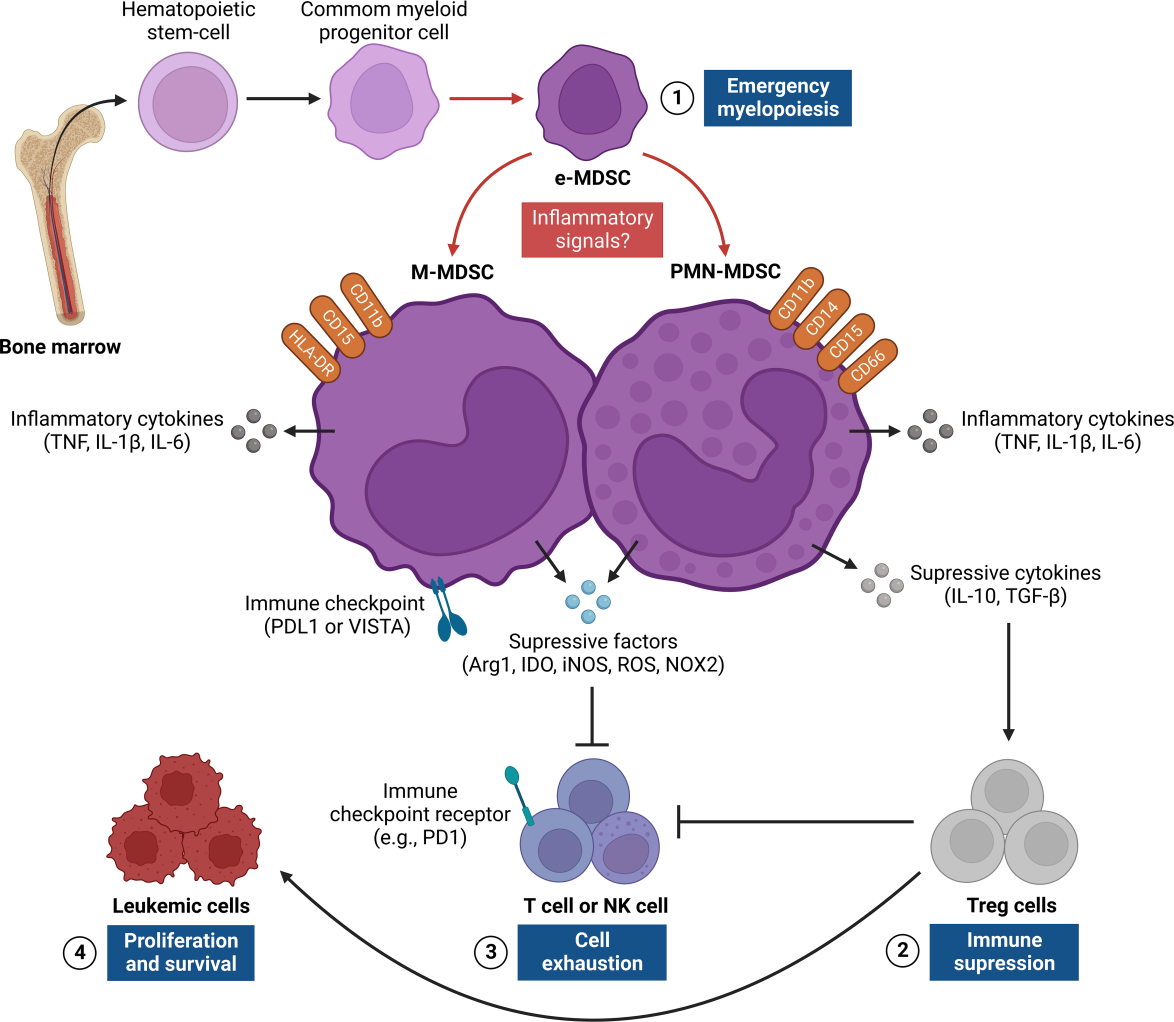
Regulation of MDSC responses in the leukemic microenvironment. The figure shows the origin and pro-tumor activities of myeloid-derived suppressor cells (MDSCs), which provide critical signals for leukemic progression. This network of interactions between MDSCs, LCs, and other immune cells [e.g., T cells, NK cells, and Treg cells] results in four main events: [1] emergence of MDSCs in bone marrow from common myeloid progenitor cells that differentiate into PMN-MDSC and M-MDSC after persistent inflammatory signals, thus characterizing a process known as “emergency myelopoiesis”; [2] activation and recruitment of Treg cells through the release of regulatory cytokines (e.g., IL-10, TGF-β), which contribute to immunosuppression of the leukemic microenvironment;[3] exhaustion of T and NK cells from different inflammatory (e.g., IL-6, TNF and IL-1β) and/or anti-inflammatory (e.g., IL-10, TGF-β, Arg1, IDO and iNOS) mediators and surface molecules (PDL1 or VISTA) expressed by MDSCs, which regulate antitumor responses;[4] LC proliferation and survival, which are a consequence of suppression of T cell and NK cell effector responses and activation of Treg cells in the leukemic microenvironment. Arg1, Arginase 1; IDO, Indoleamine; iNOS, nitric oxide synthase; M-MDSC, monocytic-MDSC; NK, Natural killer; PDL1, Programmed death-ligand 1; PMN-MDSC, polymorphic mononuclear-MDSC; TGF-β, Transforming growth factor beta; TNF, Necrosis factor tumoral; Treg, Regulatory T cells; VISTA, V-domain Ig suppressor of T cell activation.

PMN-MDSCs and M-MDSCs are activated by prolonged stimulation that is mediated by growth factors and pro-inflammatory cytokines (GM-CSF, CSF1, IL-6, and 1L-1β), as seen in conditions such as cancer, chronic infections, and autoimmune diseases (144). The stimuli that lead to their activation occur in two stages, and these are referred to as Phase 1, which is characterized by the expansion of MDSCs, and Phase 2, which is characterized by differentiation into a granulocytic or monocytic lineage (154). The expansion and activation of these cells are both dependent on transcription factors such as STAT1, STAT3, STAT6, and NF-κB, which result in the upregulation of the cytokines IL-10, TGF-β, and, in some conditions, IFN-γ; of immunosuppressive factors such as arginase 1 (ARG1), inducible nitric oxide synthase (iNOS) and reactive oxygen species (ROS); in addition to immunological checkpoint inhibitors such as Programmed death-ligand 1 (PDL1) and V-domain Ig suppressor of T cell activation (VISTA) (**Figure 3**). Together, these mediators promote anergy of cytotoxic T cells and tumor-specific T helper (Th); expansion of regulatory T cells (Treg); reprogramming of TANs and TAMs in N2 and M2, respectively; and decrease in L-arginine and L-cysteine, which are amino acids necessary for the activation and proliferation of T cells, thus collaborating in the remodeling of an immunosuppressive microenvironment that is susceptible to neoplastic progression (155–162).

In addition, MDSCs may harbor tumor-promoting functions that are independent of immune suppression, such as promoting metastasis and angiogenesis through the production of vascular endothelial growth factor (VEGF), fibroblast growth factor β (FGF-β) and matrix metalloproteinase-9 (MMP9). Furthermore, studies have described that these cells can be mobilized from the BM through G-CSF, GM-CSF, or hypoxia to metastatic environments, in which pro-inflammatory mediators, such as IL-6, TNF-α, and prostaglandin E2 (PGE2), can increase their immunosuppressive functions (16, 75, 163, 164). Thus, once activated, MDSCs become crucial factors in TME and have a significant role in the development of several solid and hematological neoplasms, in addition to being frequently associated with different stages of cancer (165–169).

In the field of acute leukemias, studies performed in pediatric patients with B-cell ALL showed a significant increase in the frequency of PMN-MDSCs, along with T reg cells in the circulation and BM compartment, which exhibited a positive association with the presence of measurable residual disease (MRD) (170). In addition, it was observed that the number of PMN-MDSCs decreased markedly in patients who went into remission, and was comparable to the control group (171). Similarly, in adult AML patients, MDSCs (CD33+ CD11b+ HLA-DRlow/neg) were significantly increased in BM and were associated with extramedullary infiltration and increased serum D-dimer concentration in plasma; however, after induction chemotherapy, there was a decrease in the frequency of these cells (172). In addition, the frequency of M-MDSCs also showed a significant increase, both in circulation and in the percentage of peripheral blood mononuclear cells (PBMCs), which was associated with a low rate of remission, high rate of relapse, and low long-term survival (173).

Regarding chronic leukemias, it was reported that patients with high-risk CML showed an increase in the frequency of PMN-MDSCs, as well as in the expression of Arg-1, which is known to inhibit T cells. In addition, PMN-MDSCs exhibited a positive upregulation for PD-L1, in conjunction with the PD-1 receptor on T cells (174). Studies also observed that the frequency of PMN-MDSCs was elevated at the time of diagnosis, and decreased to normal percentages after imatinib therapy (175). Finally, studies performed on patients with CML and CLL also observed a significant increase in the frequency of M-MDSCs at diagnosis, together with increased expression of IL-10 and TGF-β, which *in vitro* have been shown to induce T cell suppression and activation of Treg cells (174, 176, 177). In general, high frequencies of PMN-MDSCs and M-MDSCs can directly influence the clinical course of acute and chronic leukemias, thus highlighting their role as potential prognostic biomarkers and therapeutic targets in these patients (178–182).

## Concluding remarks and future perspectives

The ‘Hallmarks of Cancer’ were proposed as a set of functional capabilities acquired by human cells as they progress from normality to neoplastic growth states (183). In the most recent elaboration of this concept, after little more than a decade of incessant research into the immunobiology of cancer, the role of immune cells in the neoplastic progression is well recognized. Today, inflammation and immune evasion are considered hallmarks of cancer progression, highlighting the direct involvement of immune cells, including myeloid lineage cell populations (184). Supporting this fact, TANs, TAMs, and MDSCs represent one of the main immune infiltrates in tumor niches, and are usually associated with suppressive mechanisms that attenuate immune surveillance, cytotoxic response, and, in many cases, the success of T cell-based immunotherapies (16).

Similarly, eosinophils and basophils also infiltrate multiple tumors and are activated to regulate tumor progression, either by directly interacting with cancer cells or indirectly by modulating the TME (185–188). Platelets, small anucleate structures derived from BM megakaryocytes, have also been shown to play a broad role in tumor progression, and favor proliferation from the release of growth factors and drug resistance (189–191). In addition, they act in the formation of secondary niches through a mechanism called “cloaking”, in which platelets produce physical protection in cancer cells, thus assisting in vascular migration and extravasation to the tissues to form metastases (192–194). It is noteworthy that, although promising, these myeloid cell populations and products still present themselves as poorly recognized targets and therefore require further investigation.

**Figure 4:**
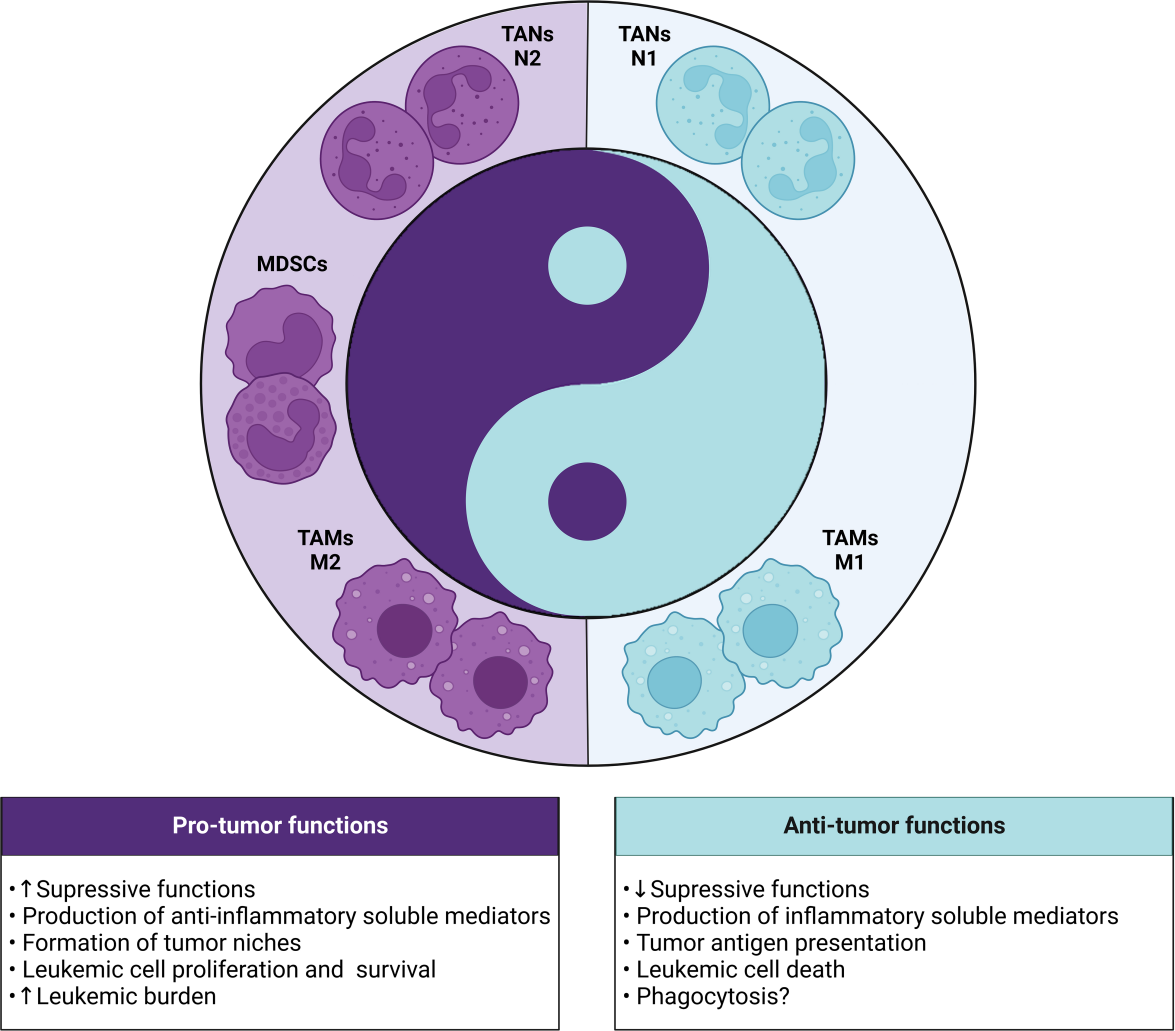
Yin-Yang with the dual role (pro and antitumor) of myeloid cells in the leukemic microenvironment. In leukemia, the different subsets of myeloid cells (TANs, TAMs and MDSCs) can exert dichotomous functions dictated by the polarization status of each cell. The pro-tumor phenotype is highlighted by the presence of N2 TANs, M2 TAMs and MDSCs, which show strong immunosuppressive activity through the production of anti-inflammatory mediators and formation of leukemic niches that contribute to the increase in tumor burden. In contrast, the antitumor phenotype involves the presence of N1 TANs and M1 TAMs that can exert protective functions through the release of cytotoxic mediators, tumor antigen presentation, death or phagocytosis of leukemic cells.

In this review, we seek to elucidate the role, mechanisms, and clinical implications of TANs, TAMs, and MDSCs in the leukemic microenvironment (**Figure 4**), as well as in the prognosis of patients with leukemia (**Table 1**). Based on the body of evidence, it is possible to suggest that the high frequency of tumor-associated neutrophils and macrophages, leaning towards an anti-inflammatory phenotype (N2 and M2, respectively), along with PMN-MDSCs and M-MDSCs could contribute to the identification of patients that are characterized by high-risk of disease at diagnosis and during treatment. In addition, several studies have highlighted the role of these cell populations as critical determinants of resistance to chemoimmunotherapy and targeted therapy, acting as a “Yin” role (5–10).

**Table 1:** Association of frequency of myeloid cell populations with the clinical implications of leukemia patients.

Myeloid cells	Leukemia subtype	Clinical implications	References
TANs^*^	**CLL^#^**	Tendency to increase the frequency of circulating immunosuppressive neutrophils (CD16^high^CD62L^dim^) according to intermediate or advanced stage of the disease.	(59)
**TAMs**	**ALL**	Increased frequency of M2 TAMs in BM¥ and extramedullary sites as an independent factor for an unfavorable prognosis.	(88, 96, 98)
	**AML**	High infiltration and mobilization of M2 TAMs in BM associated with a worse prognosis.	(88, 92, 95)
	**CLL**	Increased frequency of M2 TAMs in lymph nodes correlated with a high proliferation of LCs.	(90)
	**CML**	High frequency of M2 TAMs in BM during transition from chronic to blast phase correlated with disease progression.	(97,141)
**MDSCs**	**ALL**	Increased frequency of PMN-MDSCs in BM and blood indicative of positive MRD.	(170)
		Decreased frequency of circulating PMN-MDSCs correlated with a better prognosis.	(171)
	**AML**	High infiltration of MDSCs in the BM associated with positive MRD.	(172)
		High frequency of circulating M-MDSCs correlated with low rate of remission, disease relapse, and poor survival.	(173)
	**CLL**	Increased frequency of MDSCs correlated with greater immunosuppression and increased leukemic burden in untreated patients.	(176, 177)
	**CML**	Frequency of circulating PMN-MDSCs at diagnosis and at the end of treatment correlated with high-risk disease and poor response to chemotherapy.	(174, 175)

*TANs, tumor-associated neutrophils; TAMs, tumor-associated macrophages; MDSCs, myeloid-derived suppressor cells; ^#^CLL, chronic lymphocytic leukemia; ALL, acute lymphoblastic leukemia; AML, acute myeloid leukemia; CML, chronic myeloid leukemia; ^¥^BM, bone marrow; LCs, leukemic cells; PMN-MDSCs, polymorphonuclear MDSCs; MRD, Measurable residual disease; M-MDSCs, monocytic myeloid-derived suppressor cells.

On the other hand, we also cannot rule out the potential “Yang” role of myeloid cells in stimulating the immune response. Recent technological advances have helped the generation of genetically modified myeloid cells to enhance their antitumor properties. In summary, through genetic engineering, these cells increase the expression of cell surface receptors and antigens, as well as cytokines capable of modulating TME contributing to a pro-inflammatory environment, thus increasing the activation of cytotoxic immune cells (195). In *in vivo* studies, genetically modified myeloid cells have been shown to be able to migrate to primary tumor niches, which increases antigen presentation and IFN-γ production, promotes T cell activation, and reduces tumor burden (196, 197).

Collectively, these data indicate that the reprogramming or repolarization of myeloid cells presents itself as a promising and effective strategy, which should be explored in the context of immunotherapeutic approaches aimed at leukemias, especially considering the large cellular repertoire of the leukemic microenvironment, in addition to the intense and dynamic crosstalk between LCs and surrounding cells. Finally, it is important to highlight the scarcity of data on the dual role (Yin-Yang) of myeloid cell populations in different types of leukemia, since the characterization of the immune microenvironment of the medullary compartment can indicate relevant therapeutic targets and follow-up biomarkers of patients, in addition to providing promising immunotherapies, which would aid in controlling the disease in the long term and improving quality of life for patients.

## Author contributions

FM-G, FA-H, NA, and AC were responsible for the initial conception, project, and writing of this manuscript. FM-G, FA-H, MB, FS, CC, JM, and IF collected, analyzed, and reviewed the data. MB, FS, and FM-G designed the illustrations. FM-G, NA, FA-H, AT, AM, AT-C, and AC supervised the project development, interpreted the data, and reviewed this manuscript. All authors read, discussed the general outline of the article together and approved the final manuscript.

## Funding

This work was funded by Fundação de Amparo à Pesquisa do Estado do Amazonas (FAPEAM) (Pró-Estado Program [#002/2008, #007/2018 and #005/2019], and POSGRAD Program [#008/2021 and #005/2022]), Conselho Nacional de Desenvolvimento Científico e Tecnológico (CNPq), Coordenação de Aperfeiçoamento de Pessoal de Nível Superior (CAPES) (PROCAD-Amazônia 2018 Program-#88881.200581/2018-01). FM-G, NA, FA-H, MB, and FS have fellowships from FAPEAM, CAPES and CNPq (PhD and MSc students). JM and IF have fellowships from FAPEAM (Scientific Initiation students). AT-C and AM are level 1 and 2 research fellows from CNPq, respectively. AT-C and AC is research fellow from FAPEAM (PECTI-AM program #004/2020 and PRODOC Program #003/2022). The funders had no role in study design and decision to publish, or preparation of the manuscript.

## Acknowledgments

We would like to thank our collaborators at the HEMOAM Foundation, especially the Amazon InterScience and Leukemia Immunology Research Group; the researchers of the Post-graduate Program in Basic and Applied Immunology (UFAM) and of the Post-graduate Program in Hematology Sciences (UEA); as well as our external collaborators from the Integrated Research Group on Biomarkers (FIOCRUZ-Minas) for their critical discussions and insightful and encouraging ideas.

## Conflict of interest

The authors declare that the research was conducted in the absence of any commercial or financial relationships that could be construed as a potential conflict of interest.

## Publisher’s note

All claims expressed in this article are solely those of the authors and do not necessarily represent those of their affiliated organizations, or those of the publisher, the editors and the reviewers. Any product that may be evaluated in this article, or claim that may be made by its manufacturer, is not guaranteed or endorsed by the publisher.
